# Identifying Methods to Select and Tailor Implementation Strategies to Context-Specific Determinants in Child Mental Health Settings: A Scoping Review

**DOI:** 10.1007/s43477-023-00086-3

**Published:** 2023-05-28

**Authors:** Aksheya Sridhar, Ola Olesegun, Amy Drahota

**Affiliations:** Department of Psychology, Michigan State University, East Lansing, MI USA

**Keywords:** Implementation strategies, Strategy selection, Implementation strategy mapping methods, Scoping review, Child mental health, Implementation science

## Abstract

This scoping review describes the state of the literature regarding Implementation Strategy Mapping Methods (ISMMs) within the context of child mental health practice delivery. Goals included (a) identify and describe ISMMs addressing determinants of implementing mental health evidence-based interventions (MH-EBIs) for children and (b) describe the scope of the literature (e.g., outcomes, remaining gaps) related to identified ISMMs. Following PRISMA-ScR guidelines, 197 articles were identified. After removing 54 duplicates, 152 titles and abstracts were screened, yielding 36 articles that were screened during the full-text review. The final sample included four studies and two protocol papers (*n* = 6). A data charting codebook was developed a priori to capture relevant information (e.g., outcomes) and content analysis was utilized to synthesize findings. Six ISMMs were identified: innovation tournament, concept mapping, modified conjoint analysis, COAST-IS, focus group, and intervention mapping. ISMMs were successful in leading to the identification and selection of implementation strategies at participating organizations, and all ISMMs included stakeholders throughout these processes. Findings revealed the novelty of this research area and highlighted numerous areas for future investigation. Implications related to implementation, service, and client outcomes are discussed, including the possible impact of utilizing ISMMs to increase access to MH-EBIs for children receiving services in community settings. Overall, these findings contribute to our understanding of one of the five priority areas within implementation strategy research—enhancing methods used to design and tailor implementation strategies—by providing an overview of methods that may be utilized to facilitate MH-EBI implementation in child mental health care settings.

*Trial Registration:* Not applicable.

**Supplementary Information:**

The online version contains supplementary material available at 10.1007/s43477-023-00086-3.

## Introduction

Child mental health evidence-based interventions (MH-EBIs) improve mental and behavioral health outcomes, including reducing mental health symptoms and improving quality of life (Forman-Hoffman et al., 2017; Weisz et al., 2017). However, in usual care or community-based child MH-care settings, MH-EBIs are provided with less frequency and intensity than is recommended for effectiveness, and interventions may not be delivered with sufficient fidelity to yield positive child outcomes (Bickman, 2020; Garland et al., 2013). Resultingly, children—and especially children from marginalized backgrounds—receiving services in community-based settings may lack access to high-quality evidence-based mental health care (Alegría et al., 2010). This significant practice gap is critical to address because of the continuing rise of psychopathology among youth as well as the differential MH service availability and quality noted for marginalized youth (Alegría et al., 2010; Whitney & Peterson, 2019).

### Implementation Science

The goal of implementation science is to reduce the research-to-practice gap and improve health outcomes by increasing the uptake and utilization of EBIs within usual care settings. Implementation frameworks are hypothesized to effectively guide the adoption and sustained use of MH-EBIs within usual care settings (Proctor et al., 2009; Shelton et al., 2020; Tabak et al., 2012). This is done by utilizing rigorous methods and strategies that effectively build upon facilitating setting-specific factors and/or overcoming setting-specific hindrances to MH-EBI adoption, implementation, and sustainment.

In fact, much of the existing implementation science literature has focused on identifying and understanding determinants (i.e., barriers and facilitators) influencing MH-EBI implementation processes and outcomes across settings. Determinants may occur at multiple levels (e.g., outer context, organizational level, or inner context). For example, a common inner-context implementation barrier within community settings delivering youth MH-EBIs is the lack of organizational readiness or willingness and ability to implement a novel EBI (Scaccia et al., 2015; Stahmer & Aarons, 2009). Organizational readiness includes motivation to implement new interventions, general organizational capacity to implement new EBIs, and capacity to implement specific interventions or innovations within an organization (Aarons et al., 2011; Scaccia et al., 2015). Implementation scientists suggest that implementation determinants such as organizational readiness (or the lack thereof) be identified and addressed in order to effectively implement new EBIs within a setting (Scaccia et al., 2015). However, a paucity of literature exist guiding researchers and practitioners to effectively address implementation determinants (Cheron et al., 2019), especially as these determinants are often unique to a specific setting (Waltz et al., 2019).

### Implementation Strategies

Implementation strategies refer to systematic methods to increase the adoption, initial uptake, and sustained utilization of EBIs within novel settings (Fixsen et al., 2009; Powell et al., 2019; Proctor et al., 2013). Implementation strategies may be utilized at various phases of the implementation process to address multiple levels of implementation determinants (e.g., organizational-, client level) (Powell et al., 2019). Additionally, these strategies are believed to improve (a) implementation outcomes (e.g., improved feasibility of implementing an intervention within a setting, increased use of an intervention), (b) service outcomes (e.g., increased equity in those receiving services, improved timeliness of delivering an intervention), and (c) client outcomes (e.g., clinical improvements, improved functioning) (Proctor et al., 2011). The evidence base suggests that the use of implementation strategies is moderately effective, though several barriers (e.g., lack of consistent terminology, inconsistent reporting of the use of strategies) to evaluating implementation strategies across research exist (Kirchner et al., 2018; Leeman et al., 2017). To address these barriers, studies have focused on classifying and defining strategies to improve the use of standardized language and enhance clarity related to implementation strategies and their impact (Leeman et al., 2017; Powell et al., 2012, 2015). For example, the Expert Recommendations for Implementing Change (ERIC) studies resulted in a comprehensive list of 73 discrete implementation strategies (Powell et al., 2012, 2015). Other studies have focused on reporting and tracking the use of implementation strategies, to inform our understanding of what, how, and why strategies work within different settings (Bustos et al., 2021a; Proctor et al., 2013). However, significant gaps in our understanding of implementation strategies remain, including methods used to design and tailor strategies to best facilitate the implementation process within specific settings (Powell et al., 2019).

Overall, implementation strategies are purported to address implementation determinants to facilitate MH-EBI implementation within various settings in order to have a cascading positive impact on organizations, services, and clients (Lau et al., 2015; Proctor et al., 2011). However, given the large number of discrete implementation strategies from which to choose, it is believed that the use of tailored strategies may best facilitate MH-EBI implementation such that strategies are selected with the aim of addressing identified implementation determinants that are specific to the context in which implementation is occurring (Powell et al., 2019). While there are often similar implementation determinants across various organizations, it may be particularly effective to identify determinants that are specific and relevant to the organization in which implementation is occurring and to design, select, and tailor implementation strategies that address these unique determinants. In addition, selecting strategies that deliberately address various levels of implementation determinants may be particularly important; for example, understanding whether the implementation strategy addresses a client-level, organizational-level, or policy-level determinant may influence the effectiveness of the strategy to facilitate adoption and implementation of MH-EBIs. However, research assessing which implementation strategies may best address different levels of implementation determinants is lacking (Powell et al., 2017). One review found that tailored implementation strategies had varying levels of effectiveness when utilized within healthcare settings broadly (e.g., primary care, pharmacy) (Baker et al., 2015). Importantly, little is known about the use of tailored strategies within specific contexts, including child MH settings, despite stakeholders reporting a need for tailored implementation support when implementing child MH-EBIs in specific settings (Stadnick et al., 2022). Although studies have aimed to overcome implementation barriers to utilizing child MH practices by leveraging various discrete and multi-faceted implementation strategies (Hanson et al., 2018; Nielsen et al., 2018), the rationale behind why specific strategies were selected, how strategies address context-specific determinants, and the processes utilized to identify and select implementation strategies is often lacking. Overall, there is need for further research examining the effectiveness of tailored implementation strategies within specific contexts, including child MH-care settings. Furthermore, research outlining the processes or methods to select and tailor implementation strategies to context-specific determinants is a logical next step to enhance knowledge and use of tailored implementation strategies (Baker et al., 2015; Powell et al., 2019).

### Implementation Strategy Mapping Methods

Currently, there is a lack of consensus about best practices for selecting implementation strategies within different contexts as well as how to map these strategies onto identified implementation barriers and facilitators. Although researchers have described an overview of steps to tailor implementation strategies, few studies have empirically evaluated these processes or methods (Waltz et al., 2019). Specifically, researchers posit that setting-specific implementation determinants must first be assessed and prioritized. Thereafter, change methods (i.e., techniques that address determinants) should be identified and strategies that utilize those specific change methods should be selected to address the selected implementation determinants. Moreover, it is recommended that an implementation theory or framework guide this process in order to identify promising strategies, develop support tools, increase the chances of implementation success, and inform confirmation or refinement of the guiding theory or framework (Kirchner et al., 2018). Yet, utilizing implementation theories and frameworks to identify and understand the mechanisms of action associated with each strategy continues to be an under-researched but critical component in the process of tailoring and utilizing implementation strategies (Kirchner et al., 2018).

Overall, there remains a critical need to identify and evaluate rigorous methods for selecting, mapping, and tailoring implementation strategies to address barriers and enhance facilitators to MH-EBI implementation within specific settings, including child MH-care settings (Powell et al., 2019). Therefore, this project aimed to assess the current scope of the literature regarding Implementation Strategy Mapping Methods (ISMMs) within the context of child mental health practice delivery. ISMMs are defined as a pre-implementation approach, designed to elicit stakeholder perspectives, identify context-specific implementation determinants, and select and tailor implementation strategies that map on to each determinant, in an effort to facilitate implementation of innovations.

### Study Aims

Specifically, this scoping review aimed to

- Identify and describe ISMMs that address implementation of MH-EBIs for children, and
- Describe the scope of the existing ISMM literature within the context of child MH-EBI implementation, including identify and discuss outcomes typically measured when utilizing ISMMs, theories and frameworks guiding the use of ISMMs, and remaining gaps as well as areas for future research.

Importantly, this is the first scoping review to identify methods to select, map, and tailor implementation strategies to address specific implementation determinants within the context of child mental health practice delivery. Implementation researchers have begun to investigate specific ISMMs within other settings (e.g., behavioral health service delivery (Powell et al., 2017), healthcare (Fernandez et al., 2019), and adult mental health care delivery (Piat et al., 2021). Given the emphasis on tailoring implementation efforts to a specific setting, this scoping review seeks to contribute to the existing ISMM literature base by focusing on the use of ISMMs within the context of child mental health practice delivery specifically. Findings will advance our knowledge and understanding of methods to guide the selection and tailoring of implementation strategies, in order to inform and improve implementation efforts within child mental health service settings, with the ultimate goal of increasing access to MH-EBIs for children.

## Methods

### Literature Search Strategy

The scoping review followed PRISMA-ScR guidelines (Peters et al., 2015; Tricco et al., 2018) and involved a three-step search strategy to identify relevant articles within PsycInfo, Social Services Abstracts, and PubMed databases. Databases were identified based upon consultation with institutional librarians and experts in review methodology. Search terms were identified a priori to complete advanced searches on the databases and to identify relevant articles (Table 1). An initial search was completed in January 2021 to identify methods used to select, tailor, and map implementation strategies and to identify articles using keywords that included child MH-EBIs. A second literature search was conducted using keywords identified in the initial search. For example, methods such as concept mapping, intervention mapping, conjoint analysis, and group model building were identified during the first search, and these keywords were then utilized in the second search to identify additional relevant articles describing these methods. Keywords related to child mental health service delivery were also included. Finally, references of identified articles were reviewed to identify additional relevant literature. This search strategy was utilized six months later to identify research published since the initial search.

**Table 1:** Scoping review search terms

Search date	Search terms	Databases	Number of articles
1/6/2021	(“implementation strategy” or “Implementation strategies”) AND (“child” OR “pediatric” OR “children”) AND (“mental health service” OR “evidence-based practice” OR “evidence based intervention” or “mental health treatment”)	PsycInfo	36
		Social Services Abstracts	14
		PubMed	88
1/6/2021	(“Concept mapping” OR “conjoint analysis” OR “group model building” OR “Intervention mapping”) AND (“implementation strategy” OR “Implementation strategies”) AND (“child” OR “pediatric” OR children) AND (“mental health service” OR “evidence-based practice” OR “evidence based intervention” or “mental health treatment”)	PsycInfo	3
		Social Services Abstracts	3
		PubMed	8
1/12/2021	Reviewed references in papers identified via searches 1 and 2 to identify additional relevant literature	N/A	48
6/10/21	(“implementation strategy” or “Implementation strategies”) AND (“child” OR “pediatric” OR “children”) AND (“mental health service” OR “evidence-based practice” OR “evidence based intervention” or “mental health treatment”)	PsycInfo	4
		Social Services Abstracts	1
		PubMed	3
6/10/21	(“Concept mapping” OR “conjoint analysis” OR “group model building” OR “Intervention mapping”) AND (“implementation strategy” OR “Implementation strategies”) AND (“child” OR “pediatric” OR children) AND (“mental health service” OR “evidence-based practice” OR “evidence based intervention” or “mental health treatment”)	PsycInfo	0
		Social Services Abstracts	0
		PubMed	0

### Study Inclusion and Exclusion Criteria

Inclusion and exclusion criteria were determined prior to the title and abstract and full-text reviews (Table 2). Articles included in the title and abstract and full-text reviews met the following criteria: (a) discusses selecting/tailoring/mapping implementation strategy(ies) to address implementation determinants, (b) describes a method or process for selecting/tailoring/mapping implementation strategy(ies) to address determinants to implementation, (c) providers work with child populations (up to 18 years), and (d) mentions the implementation of mental health practices or interventions. Gray literature (e.g., unpublished articles and other publication types such as dissertations, theses, and white papers) were not excluded from our search; however, no gray literature met our inclusion criteria and thus was not included in the final set of articles. Exclusion criteria included (1) article not published in English, (2) participating providers worked exclusively with adult (18+) populations, or (3) the study focused on non-mental health practices or interventions (e.g., physical health, educational interventions). Additionally, articles were excluded during the full-text review if the article did not include a description of the population that providers were working with. All included articles were uploaded to Covidence software.

**Table 2:** Inclusion and exclusion criteria

Inclusion criteria	Exclusion criteria
Title and abstract review	
–Discusses selecting/tailoring/mapping implementation strategies to address implementation determinants –Describes a method for selecting/tailoring/mapping implementation strategies –Providers work with child populations (< 18) –Implementing mental health practices/interventions	–Article not in English –Providers who work exclusively with adult populations (> 18 years) *[If providers work with a range (e.g., 16–25) that includes under 18 years old, include]* –Non-mental health practices/interventions
Full-text review	
–Includes description of a strategy/method (e.g., concept mapping, intervention mapping) for tailoring implementation strategies to identified determinants –Delivery of MH-EBIs to child populations	–Article not in English Sample Details: –Provider who work exclusively with adult populations (18 +) *[If providers work with a range (e.g., 16–25) that includes under 18 years old, include]* –Non-mental health practices/interventions –Article does not provide information on the population of focus ISMM Details: –Article does not mention selecting/tailoring/mapping implementation strategies –Article does not describe the method for selecting/tailoring/mapping implementation strategies

**Fig. 1:**
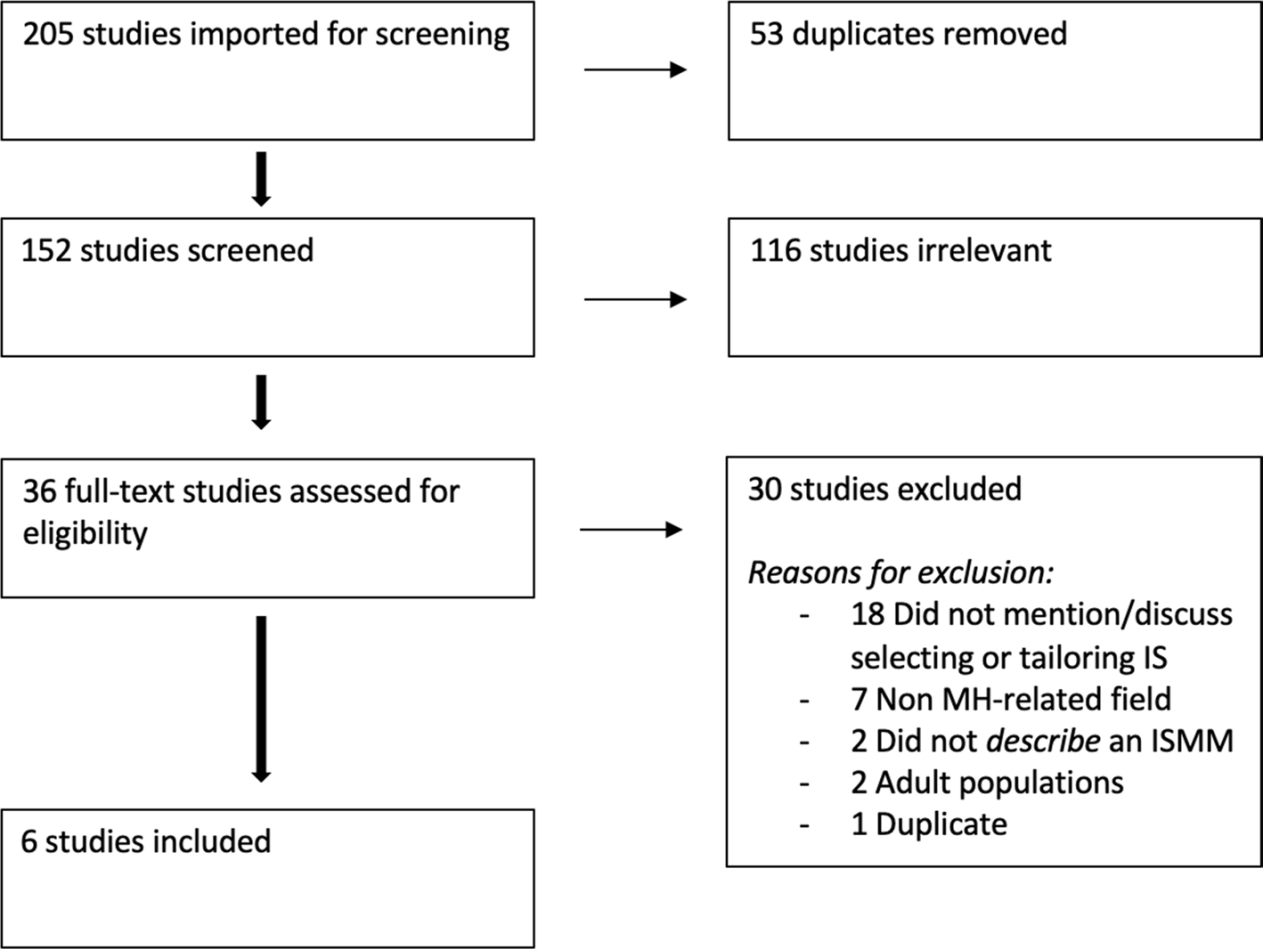
PRISMA flowchart

Following the literature search, a title and abstract review was completed during which the first and second authors individually reviewed all titles and abstracts and indicated inclusion or exclusion of the article. Next, a full-text review was completed, during which each coder independently read the article and selected to include or exclude the article using the Covidence software. When excluding an article, the first and second author noted the specific exclusion criteria supporting their decision. Reasons for excluding full-text review articles are listed in Fig. 1 (PRISMA Chart). Any discrepancies were resolved during consensus meetings.

### Data Charting and Content Analysis

Content analysis was utilized in order to determine the presence of details including citation details, participant and setting details (e.g., stakeholders involved in the studies, setting(s) in which the study was completed), descriptions and analyses of the ISMMs (e.g., name, description of the process, data analysis plans/procedures), results and findings (e.g., outcomes measured, reported findings), and implications, future directions, and limitations discussed in each study. A data charting codebook with these categories (e.g., participants and context) and codes (e.g., population, participant/stakeholder details) was developed a priori, based on the aims of the scoping review (Tables 3, 4). Emergent codes were added to the codebook iteratively based on the literature and relevance to the scoping review aims. Consistent with scoping review best practices, consensus coding was completed between the first and second author. The authors utilized MAXQDA, a qualitative coding software, throughout the data charting and content analysis process.

**Table 3:** Data charting codebook

Code	Description	Frequency
1. Citation Details		0
a. Title		9
b. Year		8
c. Author names		8
d. Journal		7
2. Participants and Context		0
a. Context/Setting	In what context or setting(s) did the study take place (e.g., schools, community clinic, organization)	16
b. Population	Who is the intervention/EBI for? (e.g., children with depression, teens with anxiety, children ages 2–5 with autism)	6
c. Intervention/EBI	What is the name of the intervention being implemented?	6
i. Intervention Purpose	What is the purpose of the intervention? (e.g., reduce depression symptoms, improve anxiety symptoms)	5
d. Participant/Stakeholder details	Who are the participants involved in selecting/tailoring/mapping the implementation strategies? (e.g., clinicians, teachers, mental health providers)	22
3. Methods		0
a. Aims/RQs	What are the study aims/research questions?	20
b. Theory/Framework	What theory or framework was used to guide use of or evaluation of the method? (e.g., CFIR, EPIS)	7
c. Defining IS	How did the authors define implementation strategies? (e.g., provided a definition, used ERIC to identify strategies)	5
d. Design	What research design was used? (e.g., qualitative study; quantitative study; mixed methods; protocol paper)	8
e. Measures and Materials	What measures were used to evaluate outcomes? What other materials were used (e.g., Software)?	26
f. DatAnalysis	What were the analyses procedures used?	15
g. Outcomes	What outcomes were measured? (e.g., effectiveness of ISMM, feasibility of ISMM)	9
4. ISMM	Name/title of ISMM	10
a. ISMM Description	Background information on the ISMM	16
b. ISMM Process	What are the steps or processes involved in the method?	14
i. # of Steps	How many steps were included in the process?	4
ii. Needs Assessment	Steps involved in a needs assessment (if this step was included)	7
iii. Identifying determinants	Process to identify barriers and facilitators (if conducted)	9
iv. Identifying implementation team	Process for identifying who will be responsible for implementation at the org (if conducted)	2
v. Creating/Selecting implementation strategies	Process for selecting implementation strategies (E.g., used ERIC to select from a list of strategies? Developed their own?)	12
vi. Sorting and ranking IS	Process for sorting/ranking IS	9
vii. Identifying mechanisms of action	Process for identifying mechanisms of action/change	2
viii. Tailoring or mapping IS	After selecting IS, how did they tailor or map the strategies onto determinants? (if a distinct step)	7
ix. Developing implementation plan/blueprint	Process for developing an implementation plan or blueprint, if described	3
x. ISMM Analysis	Analysis conducted during the ISMM process to inform other steps (e.g., analyzing determinants for ISMM)	4
xi. Data interpretation	Interpreting or evaluating data as PART OF the ISMM process/steps	6
5. Results and Findings	Results/outcomes described in the paper	0
a. Perspective re: IS	Stakeholder perspectives/perceptions of implementation strategies	9
b. Overall findings related to the ISMM	What were the findings related to the ISMM?	7
i. # of strategies identified	How many strategies were identified?	5
ii. Identifying and Addressing determinants	Did the Imp Strategies address determinants? How were determinants categorized?	5
iii. Organizing/categorizing IS	How were IS organized or categorized in the results/findings?	5
iv. Fidelity to plan/blueprint	Did they stick to the implementation plan? If not, why?	2
v. Acceptable?	Was the method acceptable/satisfactory? (if evaluated)/What aspects were acceptable?	0
vi. Useful?	Was the method useful? (if evaluated)/What aspects were useful?	1
vii. Feasible?	Was the method feasible? (if evaluated)/What aspects were feasible?	1
viii. Effective?	Was the method effective? (if evaluated)/What aspects were effective?	2
Implications	What are the implications of the results?	21
Limitations	What limitations do the authors discuss?	18
Future Directions	What future directions are discussed? What should future work focus on?	14

**Table 4:** Study characteristics

	Study design	Participants	Study setting
Innovation Tournament Sibley et al. (2021)	Design not described	Agency stakeholders (*n* = 26), parents (*n* = 226), and youth (*n* = 205)	Community Mental Health agencies (USA)
Concept Mapping Kwok et al. (2020)	Mixed methods	Speech-language pathologists (*n* = 37), clinicians (*n* = 34), policy-makers (*n* = 3), research team (*n* = 6)	Preschool Speech and Language program (Canada)
Modified Conjoint Analysis Lewis et al. (2018)	Mixed methods	Staff stakeholders (e.g., operations staff, therapists, managers) (*n* = 76)	Secure and non-secure youth residential settings (USA)
Focus Group Radovic et al. (2020)	Focus group pre-implementation study	Primary care providers (*n* = 14)	Pediatric community practices (USA)
COAST-IS Study protocol Powell et al. (2020)	Matched pair cluster randomized pilot study	Organization leaders and clinicians are expected to participate	Community mental health organizations and child advocacy centers (USA)
Intervention Mapping Study protocol Wolk et al. (2017)	Mixed methods	Stakeholder groups will include parents and providers and leaders within primary care practices	Mental Health Research Network (USA)

### Data Synthesis

After completing data charting, the first author developed summary tables on MAXQDA to review, summarize, and interpret all coded segments. Content analysis allowed for synthesizing and describing findings regarding the scope of the literature on ISMMs. Additionally, this process facilitated the identification of ISMM study limitations and remaining gaps in the extant literature.

## Results

A total of 197 articles were initially identified during the three-step search strategy: 152 articles were identified during the two initial keyword searches and an additional 48 articles were discovered through the review of references in the identified articles. Eight additional articles were identified during the search conducted six months later. Duplicate articles (*n* = 54) were removed prior to screening. In total, 152 articles were screened during the title and abstract review, with 116 of these articles being excluded. Of the 36 articles included in full-text review, 30 were excluded from data extraction for the following reasons: did not mention/discuss selecting or tailoring IS (*n* = 18), non-mental health field (*n* = 7), did not describe an ISMM (*n* = 2), adult population (*n* = 2), and one duplicate article (see Fig. 1 for PRISMA flowchart). In total, four completed studies and two protocol papers (*n* = 6) were included for data charting and content analysis. Publication dates ranged from 2017 to 2021, with half the articles (*n* = 3) published in 2020. Publication journals covered a range of topics, including implementation-focused journals, medical and clinical psychology journals, and health services research journals. Study characteristics are presented in Table 4. Scoping review findings are displayed in Online Appendix A.

### Study Design and Methods

Study designs included mixed methods (*n* = 3; Kwok et al., 2020; Lewis et al., 2018; Wolk et al., 2017), a matched pair cluster randomized pilot (*n* = 1; Powell et al., 2020), and a pre-implementation study (Radovic et al., 2020). Notably, one article (Sibley et al., 2021) did not state the study design used, as these details were reportedly described elsewhere. Quantitative measures, such as surveys and questionnaires, and qualitative measures such as interviews and focus groups were utilized across ISMM studies. Four articles (Lewis et al., 2018; Powell et al., 2020; Radovic et al., 2020; Sibley et al., 2021) utilized surveys to collect information prior to the ISMM (demographic information, stakeholder attitudes and preferences toward new evidence-based interventions/innovations, organizational readiness for change) and to collect data on the following outcomes: implementation strategy ratings (i.e., feasibility, importance), implementation outcomes (acceptability, feasibility, appropriateness, perceived utility) of the ISMM, and fidelity to the ISMM. Three ISMMs (Kwok et al., 2020; Powell et al., 2020; Wolk et al., 2017) did or will conduct interviews (semi-structured, phone) to explore stakeholders’ perspectives regarding selected implementation strategies and implementation outcomes and one study (Radovic et al., 2020) described the use of focus groups to elicit stakeholder perspectives and feedback regarding selected implementation strategies.

### Implementation Strategy Mapping Methods

Six ISMMs were identified: Collaborative Organizational Approach to Selecting and Tailoring Implementation Strategies (COAST-IS), concept mapping, focus group, innovation tournament, intervention mapping, and modified conjoint analysis. Each ISMM is described below, including details regarding: (a) steps involved in the ISMM process, (b) how implementation strategies were generated, selected, sorted or prioritized, and tailored to determinants, and (c) the analysis procedures.

#### Collaborative Organizational Approach to Selecting and Tailoring Implementation Strategies

The COAST-IS ISMM will be evaluated in community mental health organizations serving youth experiencing trauma-related emotional or behavioral difficulties, as well as within child advocacy centers (Powell et al., 2020). As proposed in the published protocol, the COAST-IS will utilize step five of intervention mapping, a method for linking implementation determinants and strategies through the identification of mechanisms of action that may impact relevant implementation outcomes. In addition, the COAST-IS plans to incorporate web-based coaching and educational sessions for organizational leaders and clinicians to guide them through the Intervention Mapping process. Like other included ISMMs, the COAST-IS will begin with a needs assessment to identify implementation determinants and performance objectives. The needs assessment will focus specifically on inner setting factors based on the EPIS model (Aarons et al., 2011). Following this, COAST-IS coaches will work with participants to identify and define implementation strategies using the ERIC compilation of implementation strategies. Participants will rate the feasibility and perceived impact of each strategy to address determinants and performance objectives. Next, to inform tailoring of implementation strategies, stakeholders will be asked to identify and explain which barriers they believe would be addressed by the selected implementation strategies. To identify implementation strategies and change methods, proposed analyses include the development of matrices that link implementation outcomes, performance objectives, and identified implementation determinants to distinguish the factors that need to be addressed to best facilitate implementation and sustainment. Finally, implementation teams and coaches will develop an implementation plan to outline the aim of implementation, scope of change, involved individuals and their responsibilities, timeline and milestones, and progress and performance measures. Implementation plans will include implementation strategy descriptions and steps to track and report on strategy use.

#### Concept Mapping

Concept mapping is a mixed-methods approach that was utilized to achieve stakeholder consensus on appropriate strategies to implement services for children receiving speech and language therapy in preschool settings (Kwok et al., 2020). This intervention was included after discussion with experts within childhood disorders and intervention science. Given the high comorbidity of communication difficulties with emotional and behavioral disorders in children, speech and language therapy is often an important component of mental health interventions for child populations (Hancock et al., 2023). Implementation determinants were identified via interviews prior to concept mapping. Participating stakeholders completed four sequential steps. First, participants generated implementation strategies through group brainstorming and then categorized and labeled strategies into similar groups. Researchers developed descriptions and summaries for strategy categories and member-checking was utilized to validate summaries. Participants then rated strategies based on perceived importance and feasibility of using the strategy. Using concept mapping software, the researchers developed pattern match graphs to explore correlations between importance and feasibility ratings, within and between stakeholder groups. The resulting graph highlighted strategies that were most feasible according to policy-makers and most important according to clinicians in order to inform prioritization of implementation strategies. Finally, researchers mapped important and feasible implementation strategies onto behavior change techniques to tailor the implementation strategies for identified determinants. Additionally, participants were asked to identify which barrier(s) they believed would be addressed by the generated implementation strategies and to identify which TDF domain was associated with each barrier. Mechanisms of action for each strategy were reviewed and discussed to determine alignment with the identified implementation barriers. Implementation strategies lacking empirical support were removed, while those supported by the literature were included as recommended implementation strategies.

#### Focus Group

A focus group approach was used in pediatric community practices across three timepoints to identify implementation determinants, select implementation strategies, obtain stakeholder feedback to adapt strategies, and elicit feedback regarding the overall approach (Radovic et al., 2020). Timepoint 1 discussions resulted in the identification of implementation determinants. At Timepoint 2, researchers provided focus groups with ideas for implementation strategies and participants provided their feedback on each proposed strategy. Lastly, researchers presented study results during Timepoint 3. Interestingly, this method did not include a process for sorting or ranking implementation strategies to reach a final consensus on top priority implementation strategies. However, this ISMM focused on stakeholder feedback and perspectives to inform the tailoring process, as stakeholders were presented with study data and a drafted list of implementation strategies and were asked to provide feedback to inform adaptations to the selected strategies. Lastly, an implementation blueprint and materials were developed to outline when and where materials should be utilized.

#### Innovation Tournament

The innovation tournament was utilized to select and tailor implementation strategies to improve the implementation of behavioral therapy for Attention Deficit/Hyperactivity Disorder (ADHD) within community mental health agencies serving youth with diverse mental health needs (Sibley et al., 2021). Participants first listed implementation barriers within their agency and were then asked to generate ideas for implementation strategies in response to open-ended prompts (e.g., strategies that would make the intervention easier to deliver). Researchers utilized the ERIC to categorize and describe each implementation strategy and then completed member-checking with stakeholders. Lastly, participants rated strategies based on importance and feasibility and ratings were analyzed to facilitate understanding of which strategies were rated as being low or high importance and low or high feasibility.

#### Intervention Mapping

Intervention mapping will be utilized within the Mental Health Research Network to increase implementation firearm safety interventions as a suicide prevention strategy (Wolk et al., 2017). Notably, this paper focuses on firearm safety intervention. The authors opted to include this paper given the focus on utilizing this intervention to prevent suicide, which was determined to be a child-focused mental health related outcome (Grossman et al., 2005). This decision was reached after discussion with implementation and intervention science experts. Similar to the COAST-IS ISMM, this study will utilize step five of the intervention mapping approach, which focuses on planning for adoption, implementation, and sustainment. Researchers plan to utilize this ISMM in conjunction with the CFIR to guide the needs assessment process and inquire about various levels of determinants (e.g., intervention level, provider level, inner context, outer context) and to identify organizational supports needed to facilitate intervention use. Following the needs assessment, researchers will translate implementation strategies into practical strategies, based on existing theory and the implementation strategy literature. To tailor these strategies, researchers propose to develop a multilevel menu of implementation strategies based on the CFIR and implementation determinants identified in the needs assessment. The generated multilevel menu will then be utilized to select the final list of implementation strategies that map onto determinants. However, the protocol paper did not describe a process for sorting, ranking, or prioritizing tailored implementation strategies and did not provide a description of the analysis plan for this ISMM.

#### Modified Conjoint Analysis

Conjoint analysis is a mixed methods approach that was modified to involve five steps for selecting and tailoring implementation strategies and utilized within secure and non-secure youth residential settings serving youth with varying mental health concerns (Lewis et al., 2018). This approach included a needs assessment, mixed-methods analysis, identifying determinants, forming an implementation team, and generating an implementation blueprint. Stakeholders began by completing a needs assessment to identify and prioritize implementation barriers based on perceived feasibility and importance to overcome or address. After prioritizing implementation determinants, participants selected implementation strategies from the ERIC and were provided the opportunity to generate additional strategies. Participants rated strategies based on their perceived feasibility and importance in addressing the implementation barriers. To tailor strategies, each implementation strategy was matched with one or more barriers based on the implementation strategy’s potential mechanism of action. This ISMM also involved the development of an implementation team at each organization that was responsible for engaging stakeholders and facilitating the implementation process. Finally, the modified conjoint analysis resulted in an implementation blueprint encompassing three phases: pre-implementation, implementation, and sustainment. Top-rated implementation strategies were organized into relevant phases within the implementation blueprint, allowing organizations to identify when each selected strategy would be utilized.

### Aims and Objectives of Included ISMM Articles

All included ISMM articles aimed or aim to identify, tailor, and match implementation strategies to context-specific determinants within an organization. One protocol paper (Powell et al., 2020) also plans to evaluate the implementation outcomes (acceptability, appropriateness, feasibility, and utility) of the ISMM, and two studies (Sibley et al., 2021; Wolk et al., 2017) aimed to illustrate the feasibility of the ISMM in order to inform future large-scale trials. Finally, four studies (Kwok et al., 2020; Lewis et al., 2018; Sibley et al., 2021; Wolk et al., 2017) included stakeholder engagement as a key aim, with these studies eliciting stakeholder perspectives to identify and explore the importance and feasibility of implementation determinants and strategies. Moreover, all studies aimed to engage multiple levels of stakeholders (e.g., agency leaders, agency staff, end-users) and two ISMMs (Kwok et al., 2020; Sibley et al., 2021) included the use of member-checking strategies throughout the process.

### Study Settings and Participants

Research examining ISMMs has or will occur in a variety of settings, including Community Mental Health (CMH) agencies providing services to youth with mental health/trauma-related needs (*n* = 2; Sibley et al., 2021; Powell et al., 2020), a preschool speech and language program (*n* = 1; Kwok et al., 2020), secure and non-secure youth residential settings (*n* = 1; Lewis et al., 2018), child advocacy centers (*n* = 1; Powell et al., 2020), pediatric community practices (*n* = 1; Radovic et al., 2020), and Mental Health Research Network systems, which include public domain research centers that involve several primary care practices (*n* = 1; Wolk et al., 2017). All six ISMMs included stakeholders who represented providers or practitioners within participating organizations (e.g., clinical supervisors, clinicians or therapists, primary care providers). Additionally, four ISMMs (Kwok et al., 2020; Lewis et al., 2018; Powell et al., 2020; Radovic et al., 2020; Wolk et al., 2017) engaged organizational leaders or managers, while two studies involved administrative staff from participating organizations in the ISMM process (Lewis et al., 2018; Sibley et al., 2021). Of the six included ISMM studies, only two (Sibley et al., 2021; Wolk et al., 2017) included end-users of the MH-EBIs (e.g., adolescents, parents of youth receiving services).

### Interventions and Populations Served

ISMM articles focused on the implementation of a range of interventions, such as therapeutic interventions for youth, outcome measurement tools, and organizational interventions. Specifically, interventions in ISMM studies included (a) STAND, an intervention that utilizes motivational interviewing and skill building to address academic and family impairment for youth with diverse mental health needs (Sibley et al., 2021), (b) Focus on the Outcomes of Communication Under Six (FOCUS), an outcome measurement tool for parents of preschool-aged children receiving speech and language therapy to track changes in child communication skills (Kwok et al., 2020), (c) Cognitive Behavior Therapy (CBT) for youth (12–18 years old) presenting with a variety of mental health concerns (e.g., anxiety, mood disorders, externalizing problems) being served in secure and non-secure residential settings (Lewis et al., 2018), (d) Supporting Our Valued Adolescents (SOVA), a technology-based intervention (i.e., social media website with educational blog posts) to increase primary care providers use of recommended treatments for adolescents with depression or anxiety (Radovic et al., 2020), (e) Trauma-Focused CBT (TF-CBT) for youth experiencing trauma-related emotional or behavioral difficulties (Powell et al., 2020), and (f) Safety Check, a suicide prevention intervention that includes counseling on firearm safety and injury prevention strategies for parents of children aged 2–11 years old (Wolk et al., 2017).

### Implementation Science Frameworks

Five studies (Kwok et al., 2020; Lewis et al., 2018; Powell et al., 2020; Radovic et al., 2020; Wolk et al., 2017) incorporated implementation frameworks to guide the project, including identifying implementation determinants, assessing determinants, and conceptualizing and measuring implementation outcomes. Frameworks included Theoretical Domains Framework (TDF, *n* = 1), Framework for Dissemination (*n* = 1), Exploration, Preparation, Implementation, and Sustainment (EPIS, *n* = 1), Implementation Outcomes Framework (*n* = 1), and the Consolidated Framework for Implementation Research (CFIR, *n* = 2).

### Study Outcomes

Completed ISMM studies primarily reported findings related to the specific determinants and implementation strategies identified and prioritized during the ISMM process. For example, stakeholders engaged in the Innovation Tournament identified several implementation strategy categories, including adapt and tailor to context, train and educate stakeholders, use of evaluative and iterative strategies, change infrastructure, engage consumers, provide interactive assistance, and support clinicians. Of the 45 strategies generated, stakeholders and the research team identified 18 important and feasible implementation strategies that were believed to address barriers (Sibley et al., 2021). The concept mapping ISMM led to the identification of 13 implementation strategies with evidentiary support to address implementation barriers. Strategies fell into six categories: resources, communication, administration fidelity, administrative logistics, user-friendliness for parents, and comprehensiveness and were perceived to address the TDF domains: beliefs about consequences and environmental context and resources (Kwok et al., 2020). In the modified Conjoint Analysis method 35 implementation strategies were identified to address the 23 implementation barriers. Strategies were organized into pre-implementation, implementation, and sustainment phases. Pre-implementation strategy categories included develop stakeholder relationships, train and educate stakeholders, support clinicians, adapt and tailor to context, use evaluative and iterative strategies, and utilize financial strategies. Implementation phase categories included train and educate stakeholders, support clinicians, change infrastructure, develop stakeholder relationships, use evaluative and iterative strategies, provide interactive assistance, adapt and tailor to context, and utilize financial strategies. Categories within the sustainment phase included train and educate stakeholders, use evaluative and iterative strategies, develop stakeholder relationships, provide interactive assistance, engage consumers, and utilize financial strategies (Lewis et al., 2018). Lastly, the focus group ISMM led to the identification of six implementation strategies: advertisement to reach a wider audience, design preferences, ability to easily demonstrate intervention in visit, physical patient reminders, PCP reminders about intervention, and electronic charting (Radovic et al., 2020). Overall, findings across the four completed ISMM studies revealed that stakeholders successfully identified numerous implementation strategies that were perceived as important and feasible in addressing implementation barriers. Moreover, implementation strategies often focused on training and resources (e.g., supporting clinicians, training stakeholders), adaptations to the context and/or the intervention, developing relationships, improving communication, and the use of strategies to evaluate implementation processes.

Outcomes related to evaluating or further understanding ISMM processes themselves were limited. One study evaluated behavioral changes at the participating organization following the ISMM process (focus group; Radovic et al., 2020). Results indicated that although participants reported motivation to utilize implementation strategies, no behavioral changes were reported as a result of engaging in the ISMM or selecting and tailoring implementation strategies (Radovic et al., 2020). Additionally, only one paper discussed the evaluation of implementation outcomes following the use of an ISMM. Powell et al. (2020) plan to measure implementation outcomes of the COAST-IS ISMM. Specifically, they will evaluate the acceptability, appropriateness, feasibility, and utility of this ISMM. No other studies included evaluated or planned to evaluate implementation outcomes related to the ISMM process.

## Discussion

### Aim 1: Identify and Describe ISMMs

Six ISMMs utilized in child mental health service settings were identified in this review: COAST-IS, concept mapping, focus group, innovation tournament, intervention mapping and modified conjoint analysis. Overall, ISMMs led to the identification of between 36 and 282 implementation strategies across the included studies. Tailoring and prioritization methods narrowed down the selected strategies to a range from 14 to 36 important and feasible implementation strategies to address identified implementation determinants within the context of child MH settings. Additionally, ISMMs involved a range of stakeholder groups, including healthcare providers, administrative staff, and end-users of the intervention.

#### Common Processes Across Methods

Several common steps and processes were noted across the ISMMs utilized in child MH settings. Five ISMMs began with a process for stakeholders to identify factors believed to influence implementation (i.e., determinants) (Kwok et al., 2020; Lewis et al., 2018; Powell et al., 2020; Radovic et al., 2020; Sibley et al., 2021), and three ISMMs reported utilizing a needs assessment to facilitate this process (Lewis et al., 2018; Powell et al., 2020; Wolk et al., 2017). However, four methods (innovation tournament, concept mapping, modified conjoint analysis, COAST-IS) reported focusing primarily on identifying implementation barriers and did not report explicitly identifying factors that facilitate implementation within the participant organizations. Notably, previous research describing steps to select and tailor implementation strategies in a broader range of settings has also primarily focused on identifying and prioritizing implementation barriers as a first step in this process (Waltz et al., 2019). Despite calls to select implementation strategies that map onto implementation determinants, which include both barriers and facilitators of implementation, these findings indicate that studies within child MH settings tend to focus on identifying and addressing implementation barriers specifically (Powell et al., 2019). Consequently, important implementation strategies that may enhance facilitators within child MH settings may not be explored during these ISMM processes and may limit the utility of these methods in this context. In contrast, the Focus Group and Intervention Mapping methods inquired about implementation determinants more broadly such that participants were asked to discuss and generate ideas about “factors that influence implementation.” It is possible that these two ISMMs may better elicit implementation strategies tailored to both addressing barriers and enhancing facilitators to implementation. However, these studies did not report explicitly asking about both barriers and facilitators; therefore, the extent to which implementation strategies were selected with both types of determinants in mind is unknown. Given that implementation determinants include factors that both hinder and facilitate implementation, it may be important to evaluate the extent to which both types of factors should be identified and prioritized to inform implementation strategy selection.

In addition to identifying implementation determinants, findings revealed that ISMM studies described various methods for selecting or generating a list of implementation strategies. Specifically, two ISMMs (modified conjoint analysis, COAST-IS) utilized the ERIC, an existing comprehensive list of implementation strategies (Lewis et al., 2018; Powell et al., 2020). In contrast, the other four ISMMs utilized stakeholder-generated ideas to develop and then select implementation strategies (Kwok et al., 2020; Radovic et al., 2020; Sibley et al., 2021; Wolk et al., 2017). Following the generation or selection of implementation strategies, half of the methods (concept mapping, modified conjoint analysis, COAST-IS) elicited stakeholder input regarding the perceived importance or impact of each implementation strategy on addressing determinants and feasibility of utilizing each implementation strategy. These processes allowed stakeholders and researchers to rank and prioritize their highest rated implementation strategies. The remaining ISMMs did not describe processes to rank and prioritize implementation strategies. Overall, findings highlighted some variation in how implementation strategies were generated or selected and prioritized across the ISMM processes utilized in child MH settings. Resultingly, best practices for engaging in ISMM steps in this context remain unknown and may be an important area for further research.

Five ISMMs described processes to tailor or adapt implementation strategies after identifying them, while the innovation tournament ISMM study reported selecting and tailoring simultaneously. These processes included steps to identify either the change method addressing a determinant or the mechanism of action of the implementation strategy. Yet, the order in which these steps occurred varied across studies. Specifically, two ISMMs focused on first identifying change methods (modified conjoint analysis) or mechanisms of action (COAST-IS) and then utilized these findings to inform strategy selection. Conversely, intervention mapping and concept mapping first selected or generated implementation strategies and then evaluated the underlying mechanisms of action for each selected strategy in order to determine the final list. Focus group and innovation tournament ISMMs did not describe processes to identify either change methods or mechanisms of action.

Overall, findings indicate that the majority of ISMMs involved identifying change methods or mechanisms of action to inform the selection and tailoring of implementation strategies, although the order in which these steps occurred varied across methods. Best practices related to the order of these various steps remain unknown and indicate possible areas for further study. Although greater understanding of both change methods and mechanisms of action are needed within existing implementation science literature, identifying change methods is considered a key step in methods to select and tailor implementation strategies (Waltz et al., 2019).

#### Additional Steps

Findings from this scoping review also revealed additional steps utilized in some ISMM processes that had not been mentioned in previous research and guidelines related to mapping methods (Waltz et al., 2019). Developing an implementation plans or blueprint outlining implementation steps, activities, and timelines was included within the modified conjoint analysis and focus group ISMMs (Lewis et al., 2018; Radovic et al., 2020), and this step is proposed as a part of the COAST-IS protocol (Powell et al., 2020). Additionally, both the modified conjoint analysis (Lewis et al., 2018) and intervention mapping (Wolk et al., 2017) ISMM included the step of identifying implementation teams that would be responsible for completing the ISMM process and facilitating child MH-EBI implementation within their organization.

#### Stakeholder Engagement and Buy-In

Notably*,* all of the ISMMs utilized in child MH settings involved or will involve diverse groups of stakeholders throughout the mapping process. Prior implementation studies have emphasized the importance of involving stakeholders who represent various roles within an organization, as well as end-users of an intervention (e.g., clients) (Beidas et al., 2019; Bustos et al., 2021b). Consistent with this previous research (Bustos et al., 2021a; Williams et al., 2020), four ISMMs described stakeholder engagement and collaboration as an integral component to ISMM processes. Specifically, innovation tournament and concept mapping methods described a member-checking step to systematically engage stakeholders. Although, the studies in this scoping review did not evaluate the effectiveness of the ISMMs or the influence of stakeholder engagement on MH-EBI implementation, it is possible that stakeholder representation and engagement may be essential to this process. Given the setting-specific knowledge that stakeholders may bring, the inclusion of diverse stakeholder groups within ISMMs may be particularly important to ensure that implementation determinants identified and prioritized are important and feasible for a particular setting, and implementation strategies selected are believed to address these determinants and are perceived as feasible. Furthermore, stakeholder buy-in, or motivation to engage in the ISMM process and to utilize the selected implementation strategies, may be critical to complete the implementation process once the ISMM process has been completed.

### Aim 2: Understand the Scope of the Literature

These scoping review findings indicate that this area of literature is nascent, as all identified studies were published during or after 2017. Further, no single child MH setting type was prevalent in included manuscripts. These findings indicate that ISMMs are hypothesized to effectively facilitate implementation efforts across a range of service delivery settings and organization types that deliver child MH-EBIs. Importantly, however, given the limited outcomes evaluated in these ISMM studies to date, further research is needed to understand the extent to which ISMMs may be feasible, acceptable, or appropriate for different types of organizations. For example, research indicates that intervention “fit” (e.g., compatibility of an intervention to the service setting) can influence the adoption and utilization of new interventions (Proctor et al., 2011). Thus, it is possible that organizational fit may be important for the use of other innovations, such as ISMMs. Further research should evaluate ISMM and organizational fit more explicitly.

This scoping review evaluated whether ISMMs typically identified a guiding theory or framework, given recommendations that theories/frameworks should guide the process of selecting and tailoring implementation strategies (Kirchner et al., 2018). All but one ISMM (innovation tournament; Sibley et al., 2021) identified an implementation framework to guide the ISMM process. Interestingly, five distinct frameworks were reported across the ISMMs, with one ISMM (COAST-IS; Powell et al., 2020) planning to utilize two D&I frameworks in tandem. Given that implementation researchers suggest selecting a theory or framework that best fits the implementation context (Kirchner et al., 2018), the use of distinct theories/frameworks across these five ISMMs may suggest that implementation researchers are indeed attempting to select theories/frameworks that best fit their study’s context. Alternatively, it is possible that these findings indicate a lack of evidence or consensus regarding which implementation theories or frameworks are best suited to guide the process of selecting and tailoring implementation strategies and mapping these strategies to implementation determinants.

#### Understanding Outcomes

The included ISMM studies primarily reported process outcomes (i.e., identifying implementation determinants; selecting implementation strategies). There were limited findings related to the effectiveness of ISMMs, stakeholder buy-in with ISMM methods, or implementation outcomes.

Only one ISMM reported effectiveness outcomes. Radovic et al. (2020) reported that no behavioral changes were observed at the organization following the focus group ISMM. This finding was attributed to additional implementation barriers (e.g., workflow barriers, lack of reminders) that arose during the implementation process. While evaluating behavioral changes following an ISMM process may provide an understanding of ISMM effectiveness, these findings highlighted methodological considerations. First, it is possible that the ISMM was not effective to facilitate implementation of child MH-EBIs. Alternatively, perhaps the implementation strategies selected in this study were not well tailored to the identified implementation determinants or participating organization. Conversely, however, this finding may indicate the importance of tracking changes to implementation determinants over time and that additional or unforeseen determinants may arise during the implementation process that were not considered during the preparation and planning phase when ISMM processes tend to occur. If this may be the case, the use of a determinant framework may be particularly useful to guide ISMM processes, as these frameworks consider how factors that influence implementation may change over the course of implementation (Nilsen, 2015).

Two studies reported stakeholder perspectives regarding the selected implementation strategies. Previous studies have emphasized exploring the extent to which stakeholders are aware of and understand the implementation processes being utilized within their organization, as well as their perspectives regarding which implementation strategies may best fit a particular setting and why strategies are believed to address specific determinants (Bustos et al., 2021a; Drahota et al., 2021; Waltz et al., 2019). Our findings indicated that although ISMM studies utilizing mixed-methods approaches collected stakeholder perspectives regarding the selected implementation strategies, none of the studies explored perspectives regarding the ISMMs specifically. As a result, our understanding of stakeholder perspectives, satisfaction with, and buy-in to ISMMs remains limited. Given the importance of stakeholder engagement as a facilitating factor for implementation efforts (Beidas et al., 2017), understanding stakeholder experiences and perceptions of the ISMM process may provide valuable information to inform and improve upon future ISMM use.

Lastly, only one ISMM protocol reported a plan to explore implementation outcomes related to ISMMs. Implementation outcomes provide an understanding of the success of the implementation effort itself, and common outcomes include feasibility (i.e., ease of use), acceptability (i.e., satisfaction), utility (i.e., usefulness), and appropriateness (i.e., fit within a setting) of a novel intervention or innovation (Proctor et al., 2011). Measuring implementation outcomes may be important for evaluating ISMMs to better understand the effectiveness and impact of systematic processes for selecting and tailoring implementation strategies and planning for MH-EBI implementation. Lack of implementation outcome measures reveals a remaining gap in the literature as there continues to be a need for understanding which ISMM may be most feasible, acceptable, useful, and appropriate for different stakeholders, end-users, organizations, and child mental health service settings (Powell et al., 2020).

### Limitations

There were several limitations to this scoping review. First, this review aimed to understand the scope of the literature on ISMMs within the specific context of child mental health services. Although this targeted focus allowed for a greater understanding of the extent to which this topic has been studied within this specific context, other ISMMs may have been identified had the focus been broader (e.g., all mental health service delivery systems, child mental, and behavioral health organizations). While the identification of only six studies and protocols may demonstrate the novelty of this field, these findings may also suggest that the eligibility and ineligibility criteria of this review limited the identification of additional ISMMs. Additionally, it is possible that additional work on this topic may have been missed based on the search criteria and limited number of databases searched. Lastly, while this review provides an overview of the six ISMMs studied within this context thus far, findings from this scoping review do not provide an indication of which ISMM may be most feasible or effective given the limited outcomes evaluated across ISMM studies.

### Future Directions

The aims of this scoping review were to identify and describe ISMMs and to explore the scope of the literature related to these processes within the context of child mental health service delivery. Findings indicate that the ISMM literature is emerging. In line with the implementation science research priority to “enhance methods for designing and tailoring implementation strategies” (Powell et al., 2019, p. 1), there are several key areas that future research could explore in order to better understand and effectively utilize ISMMs across settings.

First, several frameworks were utilized to guide the ISMM process. These were particularly integral to identifying mechanisms of action to guide the mapping process. However, methods to identify appropriate theories and frameworks or mechanisms of action were unclear in the included articles. Future research should explore whether specific theories or frameworks are particularly appropriate for the implementation strategy mapping process and the extent to which other factors (e.g., setting, clients’ characteristics) should inform theory or framework selection. Additionally, future research should expand upon how the use of theories and frameworks support the identification of mechanisms of action in order to provide guidance on systematic processes involved in selecting and tailoring implementation strategies. Greater understanding of these steps may be integral to implementation scientists and practitioners who wish to apply ISMMs within specific settings. Second, identifying implementation determinants was a common step early in the process across the ISMMs; however, most ISMMs focused specifically on the identification of implementation barriers rather than engaging stakeholders in a process to identify both barriers and facilitators. Given the importance of both of these factors as determinants in the implementation process, future research examining ISMM use should also explicitly incorporate the identification of implementation facilitators and consider how strategies may be selected to address barriers as well as enhance existing facilitators. Findings from this review also highlighted that implementation determinants may be dynamic (e.g., new determinants may arise) and that determinants may change in their importance and relevance to the implementation process. Future research should explore how ISMMs may address both types of implementation determinants and consider methods that account for changes in implementation determinants over time. Moreover**,** research is needed related to evaluating ISMM effectiveness. Specifically, implementation outcomes and longer-term outcomes, such as behavioral change (e.g., utilizing implementation strategies, greater adoption and use of innovations), may provide insight into whether ISMMs are successful in mapping implementation strategies to context-specific determinants. Finally, while findings from this review illustrated commonalities across the various ISMMs (e.g., identifying determinants), numerous processes differed across the methods. For example, different processes were utilized to inform strategy selection. For example, the ERIC compilation of implementation strategies was provided to stakeholders for strategy selection within two ISMMs, while four ISMMs utilized stakeholder-generated implementation strategies. Given the variety in processes utilized across ISMMs, several areas of future research may be important. For example, exploring implementation outcomes of ISMMs and understanding barriers or challenges to utilizing ISMMs, as well as evaluating the effectiveness (including cost-effectiveness) of the various ISMMs in comparison to other methods to select and tailor implementation strategies may be particularly important to understand which method best elicits the selection of relevant and appropriate implementation strategies and how tailoring may increase the fit and utility of implementation strategies to increase adoption, implementation, and sustained MH-EBI use.

## Conclusion

This scoping review provides an overview of the existing ISMM literature conducted within child mental health service settings. Findings highlight the novelty of research in this area; indeed, as a result of the limited scope of the literature, this review was unable to identify one particular ISMM that was most effective to facilitate behavior change, such as child MH-EBI adoption and implementation. In addition, studies rarely evaluated implementation outcomes. As a result, findings did not indicate whether any one ISMM was particularly feasible, acceptable, or useful for child mental health organizations. Nevertheless, findings do suggest that the included ISMM processes are successful in identifying and prioritizing implementation barriers and identifying and tailoring implementation strategies that are believed to address those barriers. Future research efforts in this area will provide a greater understanding of the extent to which ISMMs are effective to facilitate implementation efforts in different settings or for various interventions. Overall, ISMMs aim to improve implementation efforts by providing a systematic approach for selecting and tailoring implementation strategies so that strategies are purposefully selected to address implementation determinants that are unique to a given setting or organization. Findings from this scoping review indicate several ISMMs that have begun to be evaluated within child mental health service settings. These findings contribute to previous literature focused on implementation strategies, by providing a greater understanding of ISMMs and indicating areas for future work that aim to “enhance methods to select and tailor implementation strategies” (Powell et al., 2019, p. 1).

This scoping review contributes to our understanding of methods that may be used to address the 17-year research-to-practice gap in EBI implementation. Specifically, these findings provide implementation researchers and practitioners with valuable information regarding approaches to select implementation strategies that systematically address barriers and enhance facilitators of MH-EBI implementation within child mental health service delivery settings. Although included studies did not measure implementation, service, or client-level outcomes, it remains plausible that the use of ISMMs may improve implementation of child MH-EBIs within organizations. ISMMs may yield positive implementation-level outcomes by reducing implementation barriers and building upon implementation facilitators. Further, use of ISMMs may promote service-level outcomes, such as improved efficiency with which a child MH-EBI is implemented within an organization or increased equity in delivering child MH-EBIs. Finally, the ultimate goal of D&I science, including the use of ISMMs, is to improve clinical outcomes for end-users (e.g., youth presenting with mental health concerns). Selecting and tailoring appropriate implementation strategies within specific mental health settings may be integral to efforts aimed at increasing access to MH-EBIs for children receiving mental health services in community settings. Studies indicate that organizations providing services to children with mental health concerns experience a range of context-specific implementation barriers and facilitators. ISMMs may offer a solution to the limited availability and quality of child MH-EBIs by providing stakeholders involved in implementation with systematic steps to appropriately select and tailor relevant implementation strategies to address context-specific barriers. As a result, the use of ISMMs may have significant clinical implications if these methods successfully improve implementation efforts and sustained utilization of evidence-based mental health interventions for children who receive their services in community settings. However, as illustrated by this scoping review, the ISMM literature is nascent and these methods have yet to be studied within a number of unique contexts. As a result, the impact of utilizing ISMMs on clinical outcomes and implications for end-users remains limited and highlights an important area for future research.

## Supplementary Information

Below is the link to the electronic supplementary material.

Supplementary file1 (PDF 30 kb)
