# Supplementary material for: Identifying Methods to Select and Tailor Implementation Strategies to Context-Specific Determinants in Child Mental Health Settings: A Scoping Review
Source: Glob Implement Res Appl. 2023 May 28;3(2):212–29. doi: 10.1007/s43477-023-00086-3 (PMC10247563; doi:10.1007/s43477-023-00086-3)
Supplement: Supplementary file 1 — Supplementary file1 (PDF 30 kb) [file 43477_2023_86_MOESM1_ESM.pdf]

Appendix A. Scoping Review Findings

	Identifying Determinants	Selecting/ Generating IS	Tailoring/Adapting IS	Implementation Teams	Plan/blueprint	Stakeholder Engagement	Outcomes
**Innovation Tournament** [Sibley et al., 2021]	Participants were asked to list barriers to implementation at their agency.	Stakeholders were given prompts: "list as many (ideas) as you can think of to improve [X] barrier" and asked to generate ideas for IS.	Participants were asked to generate ideas for strategies that use the identified change methods, so that they are tailored to specific determinants.	n/a	n/a	Stakeholders: clinical staff (therapists, supervisors), organization staff (admin, office staff), and adolescent clients and their parents. Member checking conducted with parents to confirm IS.	39 strategies were identified, 18 ranked as important & feasible.
**Concept Mapping** [Kwok et al., 2020]	Barriers identified in previous study.	Participants asked to generate IS during "brainstorming" phase. Strategies were ranked by feasibility & importance.	Researchers mapped IS onto behavior change techniques. Participants asked to identify which barriers would be addressed by each strategy and identify relevant TDF domains.	n/a	n/a	Stakeholders: clinicians, program representatives, and research team. Member checking was completed after IS list was finalized.	282 strategies generated, 13 identified as important, feasible, and with evidentiary support for mechanism of action.
**Modified Conjoint Analysis** [Lewis et al., 2018]	Participants completed a needs assessment to identify and prioritize implementation barriers; barriers rated on importance and feasibility.	Strategies were selected using the ERIC. Strategies were ranked by feasibility & importance.	Each implementation strategy was matched with one or more barriers based on the implementation strategy's "potential mechanism of action".	Implementation teams were developed; included opinion leaders and champions across staff levels. Responsible for engaging stakeholders and facilitating implementation.	Developed a blueprint outlining three phases (pre-implementation, implementation, sustainment). Strategies were organized into relevant phases.	Stakeholders: operations staff, therapists, and directors/managers	23 barriers were prioritized and 36 strategies were matched to relevant barriers, and identified as important and feasible.

**Focus Group** [Radovic et al., 2020]	Determinants identified during Timepoint 1 Focus Group discussions.	Researchers provided participants with strategy ideas.	Participants were asked for feedback on each strategy during Timepoint 2 focus group.	n/a	An implementation blueprint and materials were developed; plan outlined when and where materials should be used.	Stakeholders: primary care providers, practice managers, adolescents, and young adults.	Behavioral change was measured and findings showed no changes in use of intervention. Participants were also asked for their feedback on the selected strategies.
**COAST-IS** Study protocol [Powell et al., 2020)	A needs assessment will be used to identify determinants in alignment with EPIS.	Strategies will be selected using the ERIC. Strategies will be ranked by feasibility & importance.	Participants will be asked to explain which barriers would be addressed by each strategy and why. Change methods will be identified and linked to implementation determinants and outcomes.	Implementation teams at each organization will be involved in planning implementation steps, timeline, etc.	Implementation teams and coaches will develop an implementation plan. Plan will outline aims, scope of change, individual responsibilities, timeline, progress measures, descriptions of IS, and steps to track and report IS use.	Stakeholders: organizational leaders and clinicians.	Acceptability, appropriateness, feasibility, and utility of the ISMM will be evaluated.
**Intervention Mapping** Study protocol [Wolk et al., 2017]	A needs assessment will be used to identify determinants in alignment with CFIR.	Researchers will translate IS into practical strategies based on literature.	Researchers will develop strategy men based on CFIR and determinants; menu will be used to select final IS.	n/a	n/a	Stakeholders: parents, providers, and leaders of primary care practices.	Outcomes to be measured were not described.
